# Influence of biophysical and biochemical cues on *Plasmodium* sporozoite dynamics in skin tissue

**DOI:** 10.1186/s13071-025-07065-7

**Published:** 2025-11-11

**Authors:** Nikita Gopakumar, Huang Chen, Louisa A. Messenger, Seungman Park

**Affiliations:** 1 Department of Mechanical Engineering, University of Nevada, Las Vegas, NV 89154 USA; 2 Department of Environmental and Global Health, University of Nevada, Las Vegas, NV 89154 USA; 3 Parasitology and Vector Biology (PARAVEC) Laboratory, School of Public Health, University of Nevada, Las Vegas, NV 89154 USA; 4 Interdisciplinary Biomedical Engineering Program, University of Nevada, Las Vegas, NV 89154 USA

**Keywords:** Malaria, *Plasmodium*, Sporozoites, Environmental factors, Gliding motility, Skin tissue

## Abstract

**Background:**

Malaria infection is initiated when *Plasmodium* sporozoites are injected by *Anopheles* mosquitoes into the human skin. These motile parasites must move through the dermal environment to reach blood vessels, and therefore, their ability to sense a broad range of environmental cues is critical for successful infection. The skin is a complex microenvironment with varying extracellular matrix (ECM) composition, stiffness, topography, temperature, and humidity. However, the mechanisms by which sporozoites adapt to and navigate through this landscape remain poorly understood.

**Methods:**

This review briefly summarizes literature on *Plasmodium* sporozoite motility, with an emphasis on environmental and biochemical cues that influence their behavior. Key studies utilizing intravital imaging; in vitro, in vivo, and ex vivo skin models; and engineered substrates are discussed to explore the role of physical and molecular signals on parasite dynamics.

**Results:**

This review shows that sporozoite movement is highly affected by diverse biophysical and biochemical factors, including ECM components, tissue stiffness, and surface topography. However, significant gaps remain in understanding how skin changes, especially those caused by age, inflammation, or mechanical stress, influence sporozoite navigation and successful infection.

**Conclusions:**

It is essential to understand how *Plasmodium* sporozoites sense and respond to the skin microenvironment for designing targeted interventions. Gaining deeper insight into sporozoite behavior within skin tissue—particularly the underlying signaling pathways and gene expression patterns—combined with advances in diagnostic tools and noninvasive imaging techniques targeting the initial skin-stage infection can facilitate the identification of novel therapeutic targets.

**Graphical Abstract:**

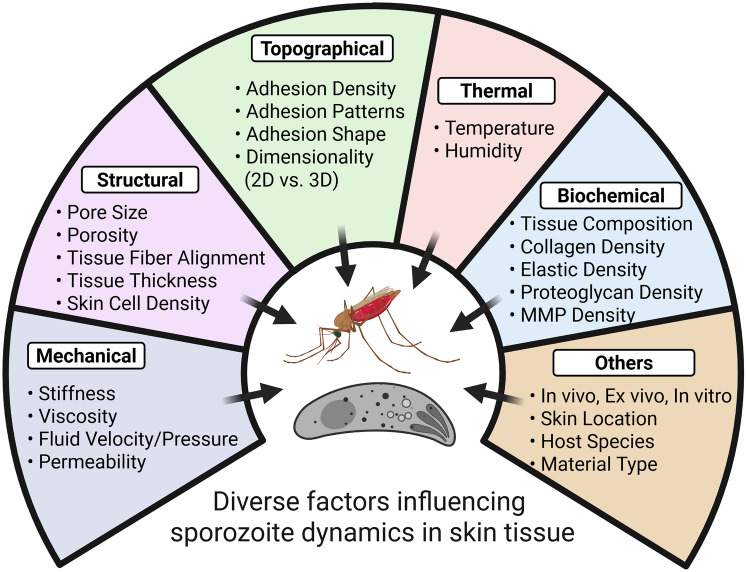

## Background

Malaria persists as a formidable global health challenge, responsible for an estimated 263 million cases and 597,000 deaths every year, across 83 countries and territories [1]. The African subcontinent endures 94% of the disease burden, attributed to environmental conditions that favor *Anopheles *(*An.*) mosquito vector breeding and the transmission of *Plasmodium *(*P.*)* falciparum*, the most lethal human malaria parasite species. The *Plasmodium* parasite life cycle is complex, involving both a vertebrate host and insect vector, where asexual and sexual reproduction occur, respectively [2–4]. Infection begins when an infected female *Anopheles* mosquito probes to take a blood meal, injecting sporozoites into the extravascular skin tissue. Due to the length of the mosquito’s proboscis (ranging between 1.5 and 3 mm) and an insertion angle of 45–60° degrees, depending on the blood meal site, the majority of sporozoites are inoculated into the dermis and, to a lesser extent, the epidermis or subcutaneous tissues [5]. Once inside the skin, sporozoites then migrate through the dermis to enter the bloodstream, which involves transversing dermal fibroblasts and endothelial cells. However, navigating dermal tissue to reach blood or lymphatic vessels poses significant physical and biological challenges, with only a small fraction of sporozoites successfully exiting the skin (~20%), rendering this stage a critical bottleneck in the infection process [6, 7]. The number of sporozoites injected during a bite may be less than 1% of those contained within the salivary glands but is strongly correlated with ruptured oocyst density and sporozoite salivary gland load, where sporozoite motility is downregulated within sporozoite aggregates in salivary cavities, potentially to regulate the number of ejectable sporozoites for optimizing parasite transmission efficiency [8–11]. Infection of *An. stephensi* with *P. yoelii* demonstrated that feeding on mice introduced a range of zero to approximately 1,300 sporozoites, with an average of ~120, and a median of 18 sporozoites per bite [12, 13]. *P. falciparum*-infected *An. stephensi* expelled a median of 136 sporozoites (interquartile range of 34–501) onto artificial skin [13]. Furthermore, the number of sporozoites transmitted is used to predict disease severity and progression; a larger inoculum of sporozoites (e.g., > 50) often leads to earlier onset of symptoms and higher parasitemia compared with lower densities (< 10), which results in delayed or incomplete liver-stage development [14]. A strong and significant correlation is evident between the salivary gland sporozoite load in mosquitoes and the size of the inoculum they deliver during a blood meal [10]. Once inside the bloodstream, the parasite moves to the liver to continue its life cycle via the hepatic stage, followed by the erythrocytic stage, which drives the clinical manifestation of malaria. Beyond serving as a physical barrier, the skin functions as an immunologically active organ, providing both naturally acquired and vaccine-induced protection during the pre-erythrocytic stage of malaria [15]. It harbors numerous resident immune cells, including dendritic cells, macrophages, and lymphocytes, which shape the early outcome of sporozoite infection. Antigen-presenting cells in skin-draining lymph nodes, particularly CD8α⁺ dendritic cells, can prime CD8⁺ T cell responses, a process mechanistically linked to the efficacy of attenuated sporozoite vaccines [16]. Antibodies further contribute by immobilizing sporozoites, blocking their vascular entry, and activating complement at the inoculation site, while skin-draining lymph nodes serve as critical sites for priming protective CD8⁺ T cell responses [17–19]. Interestingly, sporozoites can also induce local immunoregulatory pathways, such as type-I interferon-driven Tr1 responses and regulatory macrophage activity, which suppress Th1/Tfh and CD8⁺ T cell priming [20, 21]. These dual functions, protective and regulatory, of the skin immune system are directly relevant to vaccine outcomes, potentially explaining why intradermal immunization shows reduced efficacy compared with intravenous delivery in recent attenuated sporozoite vaccine trials. Therefore, more integrated studies of these dermal immune dynamics, particularly in the context of ongoing attenuated sporozoite vaccine trials, are essential for informing future malaria vaccine strategies [22, 23].

Although skin tissue acts as a gateway for malaria parasite transmission, relatively few studies have focused on understanding the mechanisms that drive dermal infection compared with liver- or blood-stage infections. In particular, the effect of skin biophysical and biochemical factors on sporozoite motility and migration patterns remains poorly understood. Motivated by this gap, we reviewed previous studies on the dynamics of *Plasmodium* sporozoites influenced by diverse environmental cues within skin tissue. We begin by exploring the mechanisms underlying sporozoite motility, with a focus on sporozoite gliding. Next, we examine metrics used to quantify sporozoite dynamics. Finally, we describe the impact of various biophysical and biochemical factors on their movement and behavior. This review aims to identify the knowledge gaps associated with *Plasmodium* skin-stage infection and highlight how improving our understanding of these parasite transmission dynamics can inform the development of novel disease prevention and treatment strategies.

### *Plasmodium* sporozoite dynamics in skin tissue

#### Principles and mechanisms of sporozoite motility in skin tissue

**Fig. 1:**
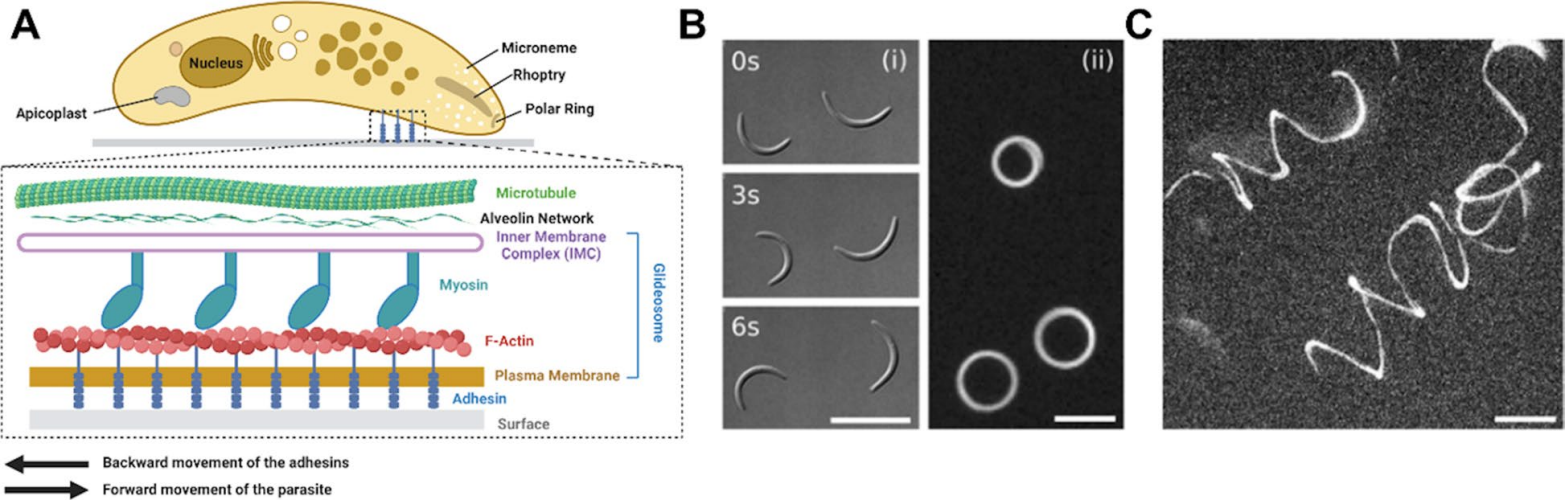
Mechanisms of gliding motility for sporozoite locomotion. **A** Diagram of a sporozoite’s internal structure and mechanism, highlighting key molecules such as actin filaments (F-actin) and myosin involved in gliding motility. The actin–myosin motor complex within the glideosome generates the force required for movement, functioning in coordination with surface adhesins. Actin filaments are translocated backward by myosin motors, which are anchored to the inner membrane complex (IMC) via light chains and gliding-associated proteins (not shown in the figure) for gliding motility. **B** Circular trajectories of two sporozoites moving counterclockwise on a 2D substrate. (i) Differential interference contrast (DIC) images at three time points (0 s, 3 s, and 6 s). Images were taken from [40]. (ii) Overlay of a time series of fluorescent images showing three gliding sporozoites. **C** Helical trajectories of two sporozoites in a three-dimensional (3D) gel. Scale bars: 10 µm. Images were taken from [40]

Sporozoites are slender, crescent-shaped cells, typically measuring approximately 10–12 μm in length and 1–2 μm in width [24, 25]. They are highly polarized cells with specialized secretory organelles located at their apical pole, specifically the micronemes and rhoptries (Fig. 1A) [26–28]. The sporozoite surface is densely populated with various surface proteins, primarily glycosylphosphatidylinositol-anchored (GPI-anchored) circumsporozoite proteins (CSP), followed by transmembrane proteins such as the thrombospondin-related anonymous protein (TRAP), membrane apical erythrocyte-binding-like (MAEBL), and others, which are involved in a range of sporozoite activities. In particular, TRAP, a micronemal protein and a member of the adhesin family, plays a crucial role in sporozoite motility, invasion, and egress; parasite lines lacking the *trap* gene still form sporozoites within oocysts but are largely incapable of entering the mosquito salivary glands [24, 29–32].

Sporozoites are fast-gliding eukaryotic cells, moving at average speeds between 1–2 μm/s, about 10 times as fast as neutrophils once inside the dermis [7, 33]. They mainly rely on actomyosin-dependent gliding motility, a unique substrate-based locomotion characterized by a lack of cilia or flagella and the absence of any overt change in cellular shape, which is different from mesenchymal or amoeboid motility observed in mammalian cells [34–38]. The gliding motility is driven by the actin–myosin complex within the glideosome, located between the plasma membrane and the inner membrane complex (Fig. 1A, inset) [28, 31]. The actin–myosin motor complex is connected to the extracellular matrix or substrate via adhesins [28]. This linkage mediates posterior movement and propels the sporozoites, a process essential for their gliding motility [28, 31]. More specifically, myosin motors, anchored to the inner membrane complex (IMC) through myosin light chains and gliding-associated proteins, pull actin filaments (F-actin) backward, while adhesins transmit the mechanical forces necessary for efficient gliding. The adhesins regulate the adhesion and deadhesion dynamics of sporozoites, with the frequency of disengagement at adhesion sites directly correlating with sporozoite speed [31, 32].

Sporozoites utilize two distinct modes of motility—avascular and perivascular—to navigate through skin tissue and reach blood vessels [6, 39]. Avascular motility facilitates rapid, less confined movement, enabling efficient dispersal across the tissue. By contrast, perivascular motility involves more curved and confined movement near blood vessels, promoting close capillary contact. Based on the two described modes, sporozoites initially appear to move randomly upon deposition in the skin. Once they encounter blood or lymphatic vessels, they transition to gliding along these structures to access the circulatory or lymphatic system [6, 7, 11]. This adaptive behavior enhances sporozoite survival and their ability to invade the bloodstream, ensuring a successful infection establishment within the host.

Interestingly, owing to their crescent shape, sporozoites exhibit different gliding motility patterns depending on two-dimensional (2D) or three-dimensional (3D) environments. On 2D substrates, the majority of sporozoites move counterclockwise in almost perfect circles [40], in a discontinuous stop-and-go; some glide with unpredictable trajectories across the substrate, and others move with a back-and-forth motion resembling a pendulum, with an amplitude of approximately one body length (Fig. 1B) [32]. By comparison, in 3D gels, sporozoites display spiral trajectories, resembling a helical corkscrew (Fig. 1C), a result of the more complex dynamics of adhesion and de-adhesion in the 3D environment [39, 40]. However, despite recent advances in understanding cell motility in 2D and 3D, further research is needed to unravel the relationship between the spatiotemporal dynamics of adhesion and deadhesion, force generation, and gliding motility in these two environments [27, 31, 41, 42].

#### Quantification of *Plasmodium* sporozoite dynamics

**Fig. 2:**
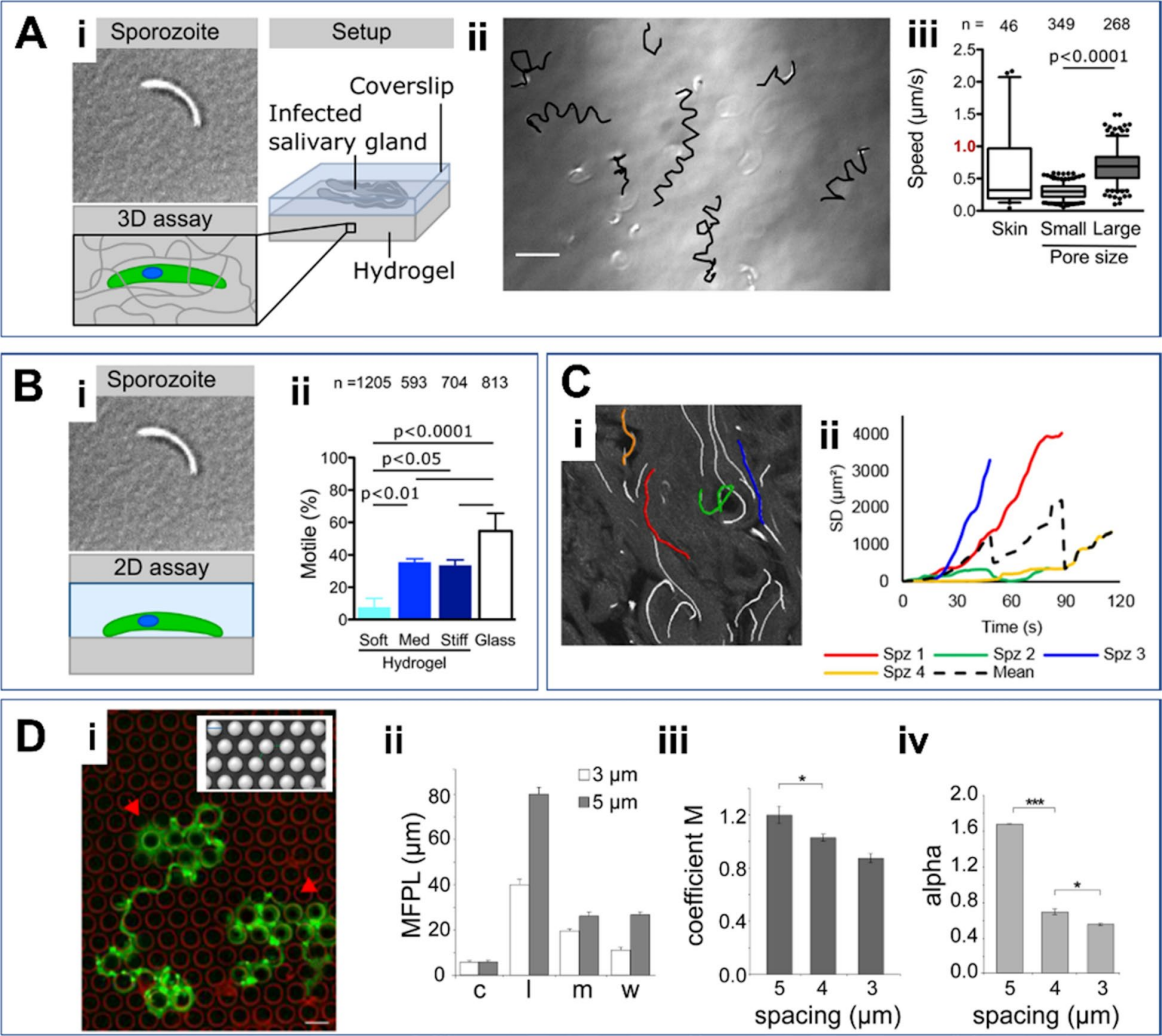
Key parameters and metrics for quantifying *Plasmodium* sporozoite dynamics. **A** Speed of sporozoites on different pore sizes and *in vivo* skin tissue. (i) Infected salivary glands positioned between a soft PA hydrogel and a glass coverslip, promoting sporozoites to move into the hydrogel. (ii) Overlay of sporozoite tracks (black) and DIC image displaying sporozoites inside a soft hydrogel at the end of the recorded image sequence. Scale bar: 20 µm. (iii) Comparison of the speeds of sporozoites *in vivo* within the skin tissue and within the soft hydrogels with small or large pores. Images were taken from [44]. **B** Quantification of the percentage of motile sporozoites under varying stiffness conditions. (i) 2D assay of sporozoite motility on planar, uncoated hydrogel, Matrigel. (ii) The percentage of motile sporozoites increases with stiffer conditions. Images were taken from [44]. **C** Mean squared displacement (MSD) of sporozoites within *in vivo* tissue. (i) Path lines of individual sporozoites on the image. (ii) Square displacements (SDs) of four different individual sporozoites over time, represented by the four different colored tracks from (i). The dotted line indicates the MSD calculated by averaging the four SDs. Images were taken from [45]. **D** Quantification of the mean free path length (MFPL) (ii), motility coefficient M (iii), and actual scaling exponent α (iv) of parasites gliding on micropillar arrays (i) of varying pillar-to-pillar distances ranging from 3 to 5 µm. Motility patterns in (ii): c, circling; l, linear; m, meandering, w, weighted. Images were taken from [39]. Scale bar: 10 µm

Understanding the behaviors and dynamics of *Plasmodium* parasites in their natural environments is essential for elucidating parasite transmission dynamics, malaria infection establishment, and the development of effective interventions. A key strategy for achieving this insight involves quantifying the motility and migration patterns of sporozoites. To date, various metrics—such as velocity, displacement, stop frequency, migration trajectories, percentage of motile sporozoites, mean squared displacement (MSD), traction force, and straightness or confinement ratio—have been utilized to characterize sporozoite movement [6, 32, 39, 43]. These analyses are conducted using diverse techniques such as single-particle tracking, digital image correlation, and manual tracking methods [6]. Velocity (or speed) and the percentage of motile sporozoites are the most commonly used metrics for quantifying the dynamics of malaria parasites in both 2D and 3D environments [32, 39, 43]. In a previous study, the percentage of motile sporozoites was analyzed under 2D conditions of varying stiffness (Fig. 2B(i)), revealing a positive correlation between substrate stiffness and the percentage of motile sporozoites (Fig. 2B(ii)). In the same study, it was observed that malaria sporozoites embedded in a 3D hydrogel (Fig. 2A(i)) exhibit helical-shaped motility (Fig. 2A(ii)) and moved faster in larger pore sizes compared with smaller ones (Fig. 2A(iii)) [44]. MSD has also been widely employed to further characterize parasite motility [6, 39, 43]. In an experimental study conducted by Winkel et al., the path lines of individual sporozoites were tracked (Fig. 2C(i)), and the square displacements (SDs) of individual sporozoites were calculated, revealing a broad range of values. These results were then used to estimate the MSD (Fig. 2C(ii)) [45]. In certain studies, researchers have employed unconventional metrics to analyze parasite movement. For instance, a previous study used mean free path length (MFPL) (Fig. 2D(ii)), motility coefficient (M) (Fig. 2D(iii)), and actual scaling exponent (α) (Fig. 2D(iv)) to quantify parasite movement within micropillar arrays (Fig. 2D(i)) with pillar-to-pillar distances ranging from 3 to 5 µm. The results revealed that MFPL varied significantly depending on the sporozoite motility patterns, such as circling (c), linear (l), meandering (m), and weighted (w) (Fig. 2D(ii)). Notably, both the motility coefficient and actual scaling exponent were found to decrease as the pillar-to-pillar distance decreased (Fig. 2D(iii–iv)) indicating a dependency on spatial constraints [39].

In addition to the aforementioned parameters, traction force, adhesion cycles, sporozoite stop frequency, diffusive behavior, and instantaneous velocity as a function of time have also been proposed as key metrics for characterizing and quantifying the dynamics of sporozoites [32, 43, 46].

#### Effects of environmental or external factors on *Plasmodium* sporozoite dynamics

The tissue environment plays a crucial role in shaping sporozoite behavior and dynamics. The environmental factors include thermal properties (e.g., temperature and humidity), structural properties (e.g., pore size, porosity, and extracellular matrix (ECM) fiber alignment), topographical properties (e.g., degree of obstacles), and mechanical properties (e.g., stiffness, relaxation time, and viscosity) [44]. Additional considerations encompass the species type (human versus animal), skin location (e.g., mouse ear versus tail in animal models), and proximity to blood vessels (near versus far from vessels). Moreover, external conditions such as experimental setup (in vivo versus in vitro), dimensionality (2D versus 3D environments), and tissue composition (e.g., collagen, polyacrylamide, and Matrigel) may significantly influence sporozoite motility and migration [6, 39, 43, 47, 48].

Thermal properties, including temperature and humidity, significantly influence sporozoite motility and malaria transmission [49–52]. This may be due to the temperature sensitivity of enzymatic activities that drive motility, underscoring the vulnerability of sporozoites to variations in their thermal environment [49]. Studies demonstrated that sporozoites exhibit a higher percentage of gliding motility at body temperature (37 °C) compared with room temperature (20–25 °C) [48, 53]. More specifically, the velocity of sporozoites nearly doubled when the temperature increased from room temperature to 37 °C, with a median velocity of 3.8 µm/s at 37 °C compared with 2.1 µm/s at room temperature [48]. In other studies, it was proposed that optimal temperatures for sporozoite development (the extrinsic incubation period; duration of sporogony) range between 23 and 27 °C [48] and that increasing temperature from 21 to 34 °C can shorten the likelihood of 50% of infected *Anopheles* to have developed sporozoites from 16.1 to 8.8 days, as suggested by mechanistic models [54]. Additional studies indicate this phenomenon may be explained by PfSET7, a histone methyltransferase, whose role in sporozoite motility is temperature-dependent, with the highest activity observed at 25 °C compared with 4 and 37 °C [55]. Humidity plays a crucial role in sporozoite dynamics; however, there is a lack of systematic studies on how moisture levels specifically affect malaria parasite motility. High moisture (the amount of water contained within the tissue) levels may help maintain a favorable environment for gliding motility on the skin, while low humidity could hinder the movement of the sporozoite by desiccating the dermis, causing reduced elastic properties of the skin, leading to a stiffer dermal environment [56, 57]. Despite this, the effects of humidity on the motility and behavior of malaria parasites have not been investigated.

**Fig. 3:**
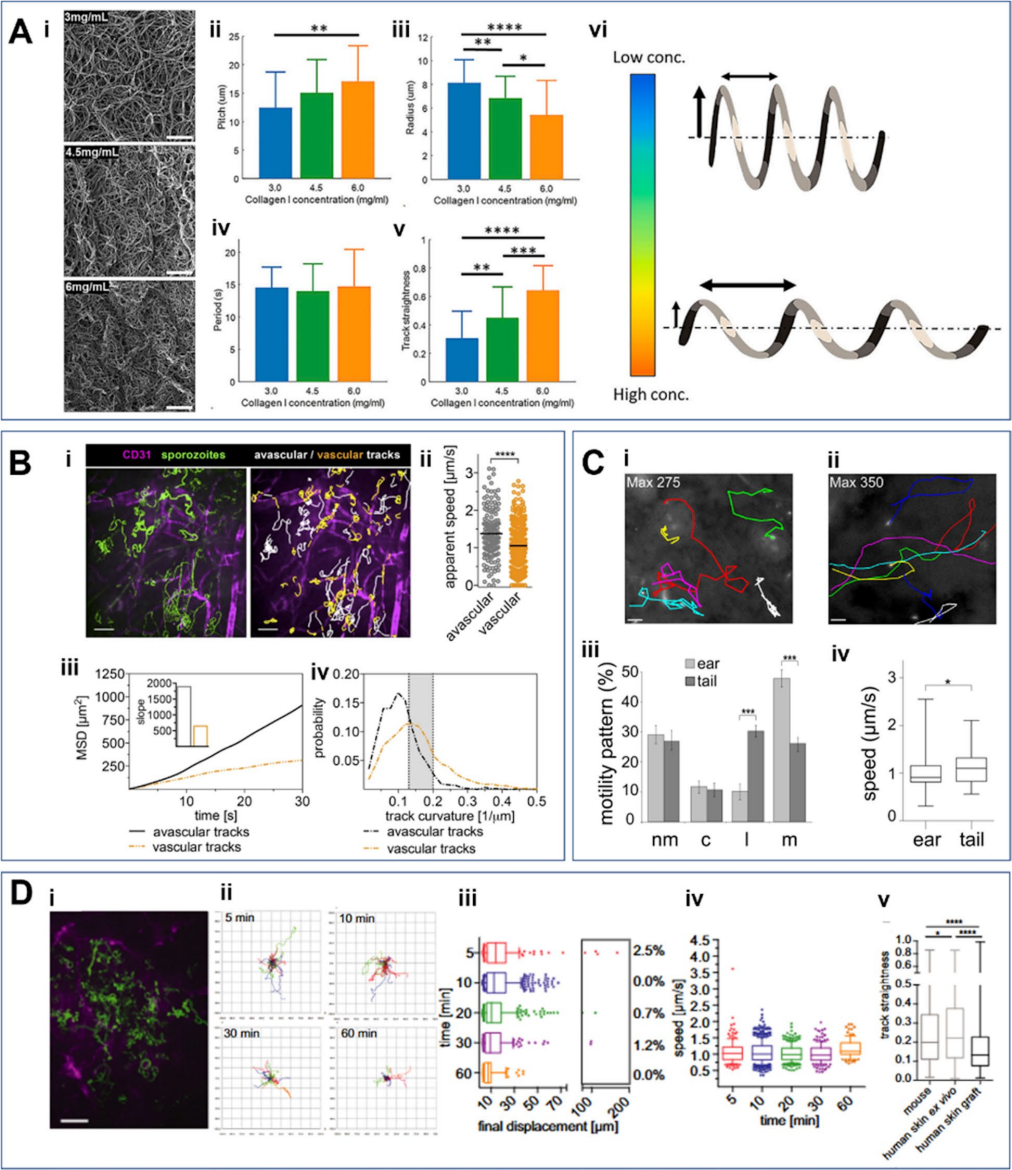
Impact of various factors on *Plasmodium* sporozoite dynamics on the dynamics of malaria parasites. **A** Effects of collagen concentration on the sporozoite dynamics. (i) Micrographs of collagen networks at collagen concentrations of 3, 4.5, and 6 mg/ml. (ii–vi) Quantified parameters, including pitch (ii), radius (iii), period (iv), and track straightness (v), based on the helical movement (vi) of sporozoites. Images were taken from Liu, [43]. **B** Effects of proximity to blood vessels on sporozoite dynamics. (i) Fluorescence images showing sporozoites (green) and blood vessels (CD31, far red). Vascular tracks (sporozoites on the vessels) are colored orange, while avascular tracks (sporozoites between vessels) are colored white. (ii–iv) Apparent speed (ii), MSD (iii), and track curvatures (iv) of sporozoites on vascular and avascular track. Images were taken from [6]. **C** Effects of skin site on malaria sporozoite dynamics. (i–ii) Path lines of sporozoites gliding in the mouse skin of the ear (i) and tail (ii). (iii) Motility patterns in the ear and tail, categorized as: nm, nonmotile; c, circling; l, linear; m, meandering. (iv) Speed of sporozoites in the ear and tail. Images were taken from [39]. **D** Effects of host species on sporozoite dynamics. (i) Trajectories of *Plasmodium falciparum* sporozoites (green) migrating in mouse dermal tissue, with CD31-labeled vessels (magenta). Scale bar: 50 µm. (ii) Tracks of sporozoites over time. (iii) Final displacement of sporozoites over time. (iv) Velocity of the sporozoites over time. (v) Straightness of sporozoite tracks (ratio of displacement to track length) in mouse dermal tissue, human skin ex vivo, and human skin grafts. Images were taken from [47]

The topographical, structural, and mechanical properties of tissues are critical factors influencing sporozoite dynamics. Regarding the topographical effects on sporozoite migration patterns, microstructured obstacle arrays with varying pillar-to-pillar spacing, ranging from 3 to 6 µm, were utilized, as shown in Fig. 2D [39]. The results revealed that arrays with larger spacing promote more circling motility patterns, along with increased velocity. Regarding dimensionality, as aforementioned, sporozoites are known to exhibit counterclockwise circular movement and a stick–slip behavior on 2D glass surfaces, relying on the formation and disassembly of discrete adhesion sites [32, 48, 58]. By contrast, within 3D hydrogels, sporozoites typically follow helical trajectories [33]. Porosity or pore size, a key tissue property, influences malaria parasite motility. Larger pore sizes generally lead to higher sporozoite speeds (Fig. 2A(iii)) [44]. Tissue stiffness is also known to enhance the dynamic behaviors of sporozoites in both 2D and 3D environments [44]. A range of biomaterials, including collagen [43], polyacrylamide [44], and Matrigel [33], have been employed to create varying mechanical conditions. Liu et al. [34] engineered different 3D stiffness conditions by adjusting collagen concentrations (Fig. 3A(i)) and analyzed the helical patterns of sporozoites embedded in the collagen gels (Fig. 3A(vi)). They found that increasing collagen concentration led to an elevated pitch (Fig. 3A(ii)) and track straightness (Fig. 3A(iii)), a decreased radius (Fig. 3A(vi)), and no change in the period (Fig. 3A(v)).

The proximity of sporozoites to blood vessels is closely linked to their movement [6]. As mentioned above, they exhibit two distinct modes of motility—avascular and perivascular—allowing them to navigate through skin tissue and reach blood vessels (Fig. 2B(i)). Avascular motility enables rapid, less confined movement (Fig. 2B(ii–iii)), facilitating efficient dispersal across the tissue. By contrast, perivascular motility involves more curved and confined movement near blood vessels, promoting close contact with capillaries (Fig. 2B(iv)). The site within the skin also plays a crucial role in influencing malaria parasite dynamics. An early study visualized the motility of sporozoites at two different mouse skin sites, the ear and tail (Fig. 2C(i–ii)), and quantified the percentage of motility patterns (Fig. 2C(iii)) and speed (Fig. 2C(iv)) at both locations [39]. Notably, they found that sporozoites moved more rapidly in the tail than in the ear. Finally, malaria sporozoite dynamics can be influenced by the host species for malaria infection. Tracking sporozoites (Fig. 2D(i–ii)) revealed that they moved in a more linear pattern in human skin tissue ex vivo (Fig. 2D(v)) compared with mouse skin tissue, as determined by quantifying displacement (Fig. 2D (iii))and speed (Fig. 2D(iv)) over time [47].

In addition to the factors mentioned above, other biophysical and biochemical factors, such as fiber alignment, interstitial fluid velocity, pressure, and tissue viscosity, should also be considered to gain a more comprehensive understanding of malaria dynamics. Exploring the interplay between external or environmental factors, structural constraints, and molecular mechanisms is essential for improving the development of novel transmission-blocking strategies, given the significant infection bottleneck experienced by *Plasmodium* sporozoites. Incorporating these elements into malaria transmission models can improve predictions of disease dynamics and inform targeted prevention strategies.

Furthermore, sporozoite motility and dynamics within the skin are regulated by factors that provide directional cues based on sulfation levels (heparan sulfate proteoglycans (HSPGs)), microtubule activity, cytoskeletal flexibility, calcium signaling, and key kinases (calcium-dependent protein kinase 6 (CDPK6), cyclic guanosine monophosphate (cGMP)-dependent protein kinase (PKG), and calcium-dependent protein kinase 4 (CDPK4)). α-tubulin expression controls microtubule nucleation, influencing motility, while inner membrane complex protein 1h (IMC1h) maintains structural integrity, ensuring efficient migration and transmission [59–62]. The most important critical factor guiding sporozoite movement is the sulfation level of host HSPGs, which provide directional cues for navigation. Sporozoites migrate efficiently through cells with low-sulfated HSPGs but are arrested in the liver sinusoids upon encountering highly sulfated HSPGs, which facilitate their transition toward hepatocyte invasion. This switch is mediated by a calcium-dependent protein kinase, which triggers the CSP, a major surface protein necessary for motility and host cell recognition. This mechanism helps sporozoite infectivity at the hepatic stage [63]. Calcium is crucial for gliding motility in *P. berghei* sporozoites, influencing various cellular processes [64]. Studies showed increased calcium levels upon activation, triggering the secretion of essential adhesins and regulating cytoskeletal interactions. This finely tuned control mechanism of calcium levels triggered adhesion secretion, helps in the gliding mechanism [64]. CDPK4 and PKG coordinate the PI-PLC/IP3 signaling pathway to regulate motility and hepatocyte invasion. PKG is essential for movement and host cell entry, while CDPK4 contributes to these processes [65]. The structural integrity and flexibility of the sporozoite cytoskeleton further influences its motility. During sporozoite gliding motility, morphological changes are driven by the dynamic reorganization of cytoskeletal structures, including the rearrangement of the pellicle and its components, such as the inner membrane complex (IMC), or alveoli, and the subpellicular microtubules (SPMTs) [66–68]. More specifically, during migration, SPMTs originate at the apical polar ring and extend toward the sporozoite’s posterior end, maintaining close association with the cytosolic face of the parasite pellicle [66]. The depletion of the protein (especially IMC1h) leads to reduced movement, abnormal morphology, and lower transmission efficiency [59, 67]. Another study showed a minimum of ten microtubules were necessary for proper sporozoite development, whereas those with fewer microtubules exhibit impaired motility and abnormal morphology [66]. Mathematical modeling revealed that α-tubulin expression levels dictate microtubule number, suggesting a high nucleation barrier, while shorter microtubules correlate with reduced motility and infectivity. These findings also emphasized the essential role of microtubule organization in *Plasmodium* development, ensuring sporozoite viability and successful transmission [66]. Taken together, the motility behavior of sporozoites is a complex process that is controlled by a tightly regulated interaction between subpellicular microtubules, the alveolin network, and actomyosin-driven forces. This regulation is likely facilitated by a combination of kinase/phosphatase signaling, calcium-dependent mechanisms, and the coupling between the membrane and cytoskeleton, ensuring that the cytoskeletal structure remains flexible enough to support motility.

From a mechanical perspective, sporozoites generate forces during gliding motility, making it essential to understand how these forces can be measured and how mechanosignaling molecules, such as the actomyosin machinery and adhesion molecules (e.g., TRAP), contribute to force generation [60]. This process is driven by the interplay between retrograde flow, force production, and adhesion dynamics [60]. Recent advancements in force measurement techniques, including traction force microscopy and optical tweezers, have enabled the quantification of the traction forces exerted by sporozoites [32, 61]. It is seen that the sporozoites can produce forces of up to 190 pN, likely involving multiple myosin motors [37, 60].

Sporozoite migration is influenced by both external forces and internal structural organization. Their elongated, polarized shape and left-handed helical motion enable them to navigate diverse environments, from mosquito salivary glands to host tissues [62]. Adhesive contacts at the poles facilitate a stick–slip movement, where stable anterior and posterior adhesions alternate with rapid detachment in the midregion [32, 37]. A recent study demonstrated that force generation is closely associated with the presence of TRAP-like protein (TLP), as sporozoites lacking TLP exhibit reduced force on their dorsal side, which shows the role of actin filament organization in force transmission and a potential therapeutic target [61]. Understanding these dynamics and mechanical properties that aid in parasite migration and facilitate host cell invasion offers valuable insights into potential targets for disrupting malaria transmission [37, 60].

**Fig. 4:**
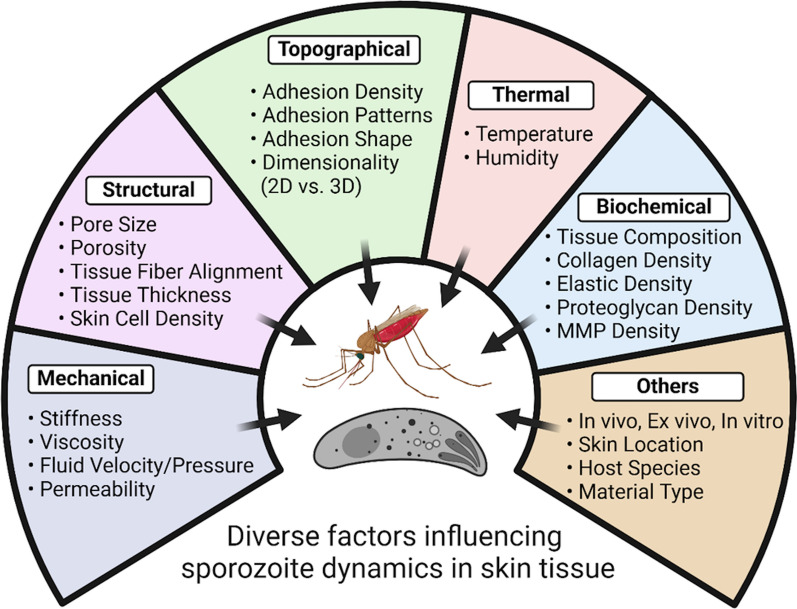
Various factors influencing the sporozoite dynamics in skin tissue. The dynamics of sporozoites can be significantly influenced by mechanical, structural, topographical, thermal, and biochemical properties and numerous other factors

Studying sporozoite dynamics and transmission presents several challenges and opens up important avenues for future research. *Plasmodium* sporozoites encounter diverse biochemical and biophysical environments, including dimensionality, stiffness, pore size, molecular/protein compositions, and surface topography, throughout their complex life cycle, necessitating adaptation to varying conditions within skin tissues that influence their gliding motility and migration patterns (Fig. 4) [39, 46].

Currently, no standardized method or set of parameters exists for effectively quantifying or characterizing sporozoite behavior within skin tissues. While velocity and displacement are the most commonly used metrics [43, 44], additional dynamic parameters, such as the confinement ratio and directional change rate, should be considered to provide a more comprehensive and accurate description of sporozoite dynamics in both 2D and 3D environments. Moreover, the mechanisms underlying the transition between sporozoite motility modes remain poorly understood [45]. Specifically, the mechanisms underlying the occurrence of a low-speed, circular-moving, subdiffusive state near blood vessels and a high-speed, forward-moving, superdiffusive state farther from blood vessels warrant further investigation [7, 69]. To unravel this phenomenon, it is crucial to illuminate the relationship between TRAP adhesion proteins, actomyosin motors, and sporozoite motility [30, 42, 70].

In vivo imaging has been frequently used to characterize the motility of malaria sporozoites in animal models such as mice [6, 7, 11, 39, 47]. However, the use of animal models is associated with significant drawbacks, including high costs, time-consuming protocols, and labor-intensive processes [71]. To address these limitations, various biopolymers and hydrogels, such as collagen, polyacrylamide, and Matrigel, have been utilized as alternative skin model systems for malaria research [43, 44]. These materials offer a key advantage in their tunability; by adjusting polymer or crosslinker concentrations, it is possible to engineer desirable physical properties such as stiffness, viscosity, fiber alignment/architecture, and pore size/porosity [44, 72–75]. This flexibility enables the study of how specific physiologically relevant parameters influence the motility of *Plasmodium* sporozoites. Most existing in vitro biomaterials used in previous studies lack components such as proteoglycans, fibronectin, and elastin, which are essential in skin physiology [43, 76, 77]. To develop a more realistic and comprehensive skin model, these components must be incorporated, allowing for improved biochemical and biophysical mimicry of skin tissue [6, 10, 43, 45, 47, 48, 76, 78–80]. Such advancements can enable the generation of more physiologically relevant and accurate experimental outcomes.

Addressing these research gaps will not only enhance our understanding of sporozoite behavior but also advance malaria treatment and prevention efforts significantly. Specifically, gaining insights into sporozoite dynamics under varying environmental conditions could facilitate the development of novel transmission-blocking antibodies or vaccines to disrupt or prevent parasite migration, ultimately strengthening malaria control strategies.

## Conclusions

While existing antimalarial drugs such as atovaquone and artemisinin target the later stages of the parasite lifecycle, new strategies that might potentially neutralize sporozoites during the initial skin stage could help prevent their entry into the bloodstream [81]. Vaccines targeting the sporozoite’s ability to infect the skin present a promising approach, for example, by inducing antibodies that bind to major surface proteins, thereby inhibiting or disrupting the movement of the sporozoite [82]. Another approach could disrupt key kinases, such as CDPK4 and PKG, which would impair the sporozoite migration, making them potential therapeutic targets for malaria prevention [65].

Although numerous metrics and methods have been proposed to characterize and quantify sporozoite motility and migration patterns, no definitive metric fully captures sporozoite behavior, necessitating multiple combined parameters for a more comprehensive understanding. Furthermore, various environmental factors, including biophysical, thermal, and biochemical properties, can affect sporozoite dynamics, yet their effects remain largely unexplored. Bioengineered skin models such as skin explants, skin-on-a-chip systems, and skin organoids help fill the critical knowledge gap of host–parasite interaction by offering anatomically relevant, reproducible, and accessible systems that can transform parasitology research and therapeutic development [83]. A deeper understanding of sporozoite behavior within skin tissue, including the associated signaling pathways and gene expression, along with advancements in diagnostic tools and noninvasive imaging techniques targeting the initial skin-stage infection, may contribute to the development of novel therapeutic targets.

## Data Availability

All data generated or analyzed during this study are included in this published article.

## Abbreviations

CSP: Circumsporozoite protein
MFPL: Mean free path length
MSD: Mean squared displacement
MAEBL: Membrane apical erythrocyte-binding-like
TRAP: Thrombospondin-related anonymous protein
